# Supplementary material for: Body fat, skin tone, and the accuracy of smartwatch caloric expenditure estimates
Source: PLoS One. 2026 Jul 29;21(7):e0353261. doi: 10.1371/journal.pone.0353261 (PMC13419227; doi:10.1371/journal.pone.0353261)
Supplement: Attachment — Submitted filename: reviwers comments PLOS ONE R2 PONE-D-26-00179.docx [file pone.0353261.s003.docx]

**Ref: PONE-D-26-00179 revision (R2)**

**Body Fat, Skin Tone, and the Accuracy of Smartwatch Caloric Expenditure Estimates**

PLOS One

**Editor's comments**

**Thank you for submitting the revised manuscript. It seems that some sections may still require additional revision. Please carefully address the reviewers' comments and resubmit the manuscript at your convenience.**

Response: We thank the Editor and the reviewer for the opportunity to provide this minor revision. We appreciate the recognition of the study’s potential contribution and have carefully considered the reviewer’s comment. The manuscript has been revised to address the suggested improvement. We detail the resulting changes below and highlight them in the manuscript.

Reviewers' comments:

Reviewer's Responses to Questions

**Comments to the Author**

1. If the authors have adequately addressed your comments raised in a previous round of review and you feel that this manuscript is now acceptable for publication, you may indicate that here to bypass the “Comments to the Author” section, enter your conflict of interest statement in the “Confidential to Editor” section, and submit your "Accept" recommendation.

Reviewer #3: All comments have been addressed

Response: Thank you.

2. Is the manuscript technically sound, and do the data support the conclusions?

The manuscript must describe a technically sound piece of scientific research with data that supports the conclusions. Experiments must have been conducted rigorously, with appropriate controls, replication, and sample sizes. The conclusions must be drawn appropriately based on the data presented.

Reviewer #3: Yes

Response: Thank you.

3. Has the statistical analysis been performed appropriately and rigorously?

Reviewer #3: Yes

Response: Thank you.

4. Have the authors made all data underlying the findings in their manuscript fully available?

The [PLOS Data policy](https://urldefense.com/v3/__http:/track.editorialmanager.com/CL0/http:*2F*2Fwww.plosone.org*2Fstatic*2Fpolicies.action*23sharing/1/010f019eb9a1975a-8ad4da67-d9f7-4fa2-b8d7-80fdcc4a17aa-000000/lmT-H7A33PSj3WFNBBQV0sUAjO36oR2c2NdbN2HeP8w=258__;JSUlJSU!!FjuHKAHQs5udqho!OlzzU3RtB92bbFBkd6W85AHeNluS2lg_L7dzrHFKRjyXBwcGKb3s7Sz7g9xlq761l-wwTHc3YSrUXQTX$) requires authors to make all data underlying the findings described in their manuscript fully available without restriction, with rare exception (please refer to the Data Availability Statement in the manuscript PDF file). The data should be provided as part of the manuscript or its supporting information, or deposited to a public repository. For example, in addition to summary statistics, the data points behind means, medians and variance measures should be available. If there are restrictions on publicly sharing data—e.g. participant privacy or use of data from a third party—those must be specified.

Reviewer #3: Yes

Response: Thank you.

5. Is the manuscript presented in an intelligible fashion and written in standard English?

PLOS ONE does not copyedit accepted manuscripts, so the language in submitted articles must be clear, correct, and unambiguous. Any typographical or grammatical errors should be corrected at revision, so please note any specific errors here.

Reviewer #3: Yes

Response: Thank you.

6. Review Comments to the Author

Please use the space provided to explain your answers to the questions above. You may also include additional comments for the author, including concerns about dual publication, research ethics, or publication ethics. (Please upload your review as an attachment if it exceeds 20,000 characters)

**Reviewer #3:** I believe the authors have already successfully addressed the comments raised by previous reviewers, and the manuscript is suitable for publication. I would only suggest briefly indicating the BMI levels/cut-offs according to WHO classification standards for clarity.

Response: We have revised this sentence to indicate the WHO categorization of BMI for obesity as 30.0 kg/m^2^.
