# Supplementary material for: Body fat, skin tone, and the accuracy of smartwatch caloric expenditure estimates
Source: PLoS One. 2026 Jul 29;21(7):e0353261. doi: 10.1371/journal.pone.0353261 (PMC13419227; doi:10.1371/journal.pone.0353261)
Supplement: Attachment — Submitted filename: reviwers comments PLOS ONE R1 PONE-D-26-00179.docx [file pone.0353261.s002.docx]

**Ref: PONE-D-26-00179 revision (R1)**

**Body Fat, Skin Tone, and the Accuracy of Smartwatch Caloric Expenditure Estimates**

PLOS One

**Editor's comments**

**Thank you for submitting your manuscript to PLOS ONE. After careful consideration, we feel that it has merit but does not fully meet PLOS ONE’s publication criteria as it currently stands. Therefore, we invite you to submit a revised version of the manuscript that addresses the points raised during the review process.**

Response: We thank the Editor and both reviewers for the thoughtful assessment of our manuscript and for the opportunity to revise and improve the work. We appreciate the recognition of the study’s potential contribution and have carefully considered all editor and reviewer comments. The manuscript has been revised extensively to address the suggested improvements and to strengthen clarity, methodological rigor, and interpretation throughout. We detail the resulting changes below and highlight them in the manuscript.

**1.** **Please ensure that your manuscript meets PLOS ONE's style requirements, including those for file naming.**

Response: We have reformatted the manuscript in line with the current PLOS ONE templates. Separate files for the main text, and figures have been prepared and named according to the journal's conventions.

**2. The ethical approval number(s) listed in the manuscript and submission metadata does not match the approval number on the ethical approval document you provided.**

Response: Throughout the manuscript and in the submission metadata we now use the protocol number IRB‑22‑0471, consistent with the approval letter issued by the IRB on 12 October 2022.

**3. Funding information should not appear in the Acknowledgments section or other areas of your manuscript.**

Response: All funding information has been confined to the Funding Statement within the submission system. The acknowledgments now solely thank the research assistants and study participants.

**4. Your abstract cannot contain citations.**

Response: The abstract has been rewritten for clarity and now contains no citations. Prior work is referenced in the introduction instead.

**5. If the reviewer comments include a recommendation to cite specific previously published works, please review and evaluate these publications to determine whether they are relevant.**

Response: There were no directly suggested references.

**Reviewer #1**

Response: Thank you for your thorough and well-structured review. We have addressed each comment and highlighted the respective changes in the revised manuscript.

**Title and Abstract**

1. **(Lines 50–52): The wording is unclear and would benefit from rephrasing for clarity and flow.**

Response: We rewrote the abstract (Lines 50–52) for clarity.

2. **Physical activity energy expenditure (PAEE) should be defined in full at first mention in the abstract.**

Response: Physical activity energy expenditure (PAEE) is now defined on first use

3. **The primary objective is not clearly stated in the abstract and should be explicitly articulated.**

Response: The primary objective is explicitly stated: “The primary objective of this study was to quantify the accuracy of smartwatch-derived physical activity energy expenditure (PAEE) estimates relative to indirect calorimetry and to examine whether error varies as a function of device brand, body fat percentage, and skin tone.”

4. **References are generally not included in abstracts; prior work should be described using general phrasing without citation.**

Response: All citations were removed from the abstract as per journal policy.

**Methods**

**Ethics and Recruitment**

5. **There is a discrepancy between the stated ethics approval date (2024) and the reported recruitment start date (January 2023), which requires clarification.**

Response: The Methods now note that IRB approval was granted on 12 October 2022 and that recruitment began in January 2023 after approval.

6. **The manuscript should explicitly state that participation was voluntary and that alternative assignments were offered to minimise the risk of coercion.**

Response: We added a statement that participation was voluntary and that course credit–seeking students were offered alternative assignments.

7. **The recruitment strategy should be clearly planned and described a priori; changes to recruitment methods over time should be justified if applicable.**

Response: The recruitment strategy (campus announcements and flyers) was planned in advance and this is now described in the Procedures section, there are no deviations to report.

**Participants**

8. **The method used to stratify body mass index (BMI) is not described and should be specified.**

Response: BMI strata are now described using the World Health Organization cut‑points, and this stratification is only used to characterise the sample.

9. **Demographic and baseline characteristics should be reported in the Results section rather than in the Participants subsection of the Methods.**

Response: Baseline characteristics (age, sex, BMI, body‑fat percentage, Fitzpatrick skin type) have been moved to the beginning of the Results section.

**Study Design**

10. **The design is acceptable; however, the authors should briefly clarify the between-subject and within-subject factors of the mixed factorial design and describe the randomisation of device placement conditions.**

Response: The design is now described as a four‑level within‑subject factor of device (Apple, Garmin, Samsung, Fitbit) crossed with continuous body fat percentage and categorical Fitzpatrick skin type. Device placement was randomized across wrists using a Latin‑square schedule, which is now detailed in the Design and Measures section.

**Measures**

11. **The description of wearable devices is generally clear; however, the authors should clarify wrist placement, ordering of the five devices, and address potential interference from simultaneous wear.**

Response: We have clarified the randomization procedure for the devices, included additional details about device placement in the measures section, and previously conducted a sensitivity analysis to search for potential interference (briefly reported in text and fully shared in the OSF registry analysis), which found no evidence of such interference.

12. **Ensure that this section is limited to methodological descriptions and does not include results.**

Response: We have moved any results to the results section as appropriate.

**Data Analysis**

13. **Line 206 should clearly state whether the COSMED K5 was used as the criterion reference.**

Response: We have modified the language to explicitly state that the COSMED K5 served as the criterion reference.

14. **Five devices are described earlier, yet the statistical models refer to device as having four levels; this discrepancy must be clarified.**

Response: The Empatica E4 did not report caloric expenditure and was therefore excluded from analyses as indicated and now clarified in the measures section, leaving four device levels. Originally, the Empatica was included for its ability to provide open-source access to the PPG waveform; software support for the device ended before analysis.

15. **The inclusion of all pairwise interaction terms should be briefly justified or stated as prespecified.**

Response: All pairwise interactions among device, body‑fat percentage (z‑scored), and Fitzpatrick type were prespecified and this is now explained.

16. **A short explanation should be added to justify the log-transformation of AE and APE.**

Response: Log‑transformation of absolute error (AE) and absolute percentage error (APE) is justified to normalize right‑skewed distributions, this has been explained in text.

17. **Truncated sentences should be corrected and abbreviated terms (for example, “Fitz”) written in full.**

Response: Abbreviations such as ‘Fitz’ have been replaced with full terms and the truncated sentences have been corrected.

18. **A brief justification for the inclusion of one-sample t-tests alongside mixed-effects models would improve clarity.**

Response: One‑sample t‑tests are included to assess whether mean device bias differs from zero prior to mixed‑effects modelling, as clarified in the Data Analysis section.

**Results**

19. **Demographic and baseline participant data should be presented in the Results section.**

Response: Demographic and baseline characteristics now open the Results section.

20. **While the Results collectively address device- and adiposity-related PAEE error, the authors should ensure that all prespecified objectives, particularly those related to skin tone and the criterion reference, are clearly reflected in the Results structure and subsection headings.**

Response: The Results are have been reorganized into subsections which contain clear outcomes related to prespecified objectives (device error metric, body fat effects, skin tone effects and criterion reference performance) to ensure that each is clearly addressed.

**Discussion**

21. **The Discussion is well written and appropriately interprets the findings in light of the study design and analyses. Minor revisions are recommended to more clearly contextualise subgroup limitations and to maintain appropriate caution in device-level comparisons.**

Response: Thank you for this compliment. We added a sentnce noting that the sample comprised primarily Hispanic adults with Fitzpatrick types III–V, and that the null skin‑tone finding should be interpreted cautiously due to limited statistical power.

**Reviewer #2**

This manuscript presents a well-designed validation study examining the accuracy of physical activity energy expenditure (PAEE) estimates from four widely used wrist-worn wearables against indirect calorimetry during a controlled cycling protocol. The study is preregistered, employs an appropriate criterion measure (COSMED K5), applies mixed-effects modeling, and makes data and code publicly available via OSF. The focus on body fat percentage (BF%) as a moderator of device error is a clear strength and addresses an important gap in the literature.
However, several methodological and interpretational issues require clarification and additional analyses before the conclusions can be fully supported.

Response: Thank you for your review and the recognition of the strengths of this research as well as your thorough critique. We have addressed each comment and highlighted the respective changes in the revised manuscript.

**Major concerns:**

1. **Clarification of PAEE definition and analyzed time window**

**The protocol includes rest, exercise, and recovery phases, but it is not fully clear which segments were included in the PAEE error calculations. Please explicitly specify whether error metrics (Bias, AE, APE) were computed over the entire session, the exercise phase only, or separately by intensity. In addition, clarify whether devices reported “active” versus “total” calories and how this was harmonized across brands. These details are critical, as low-intensity periods may disproportionately affect APE and between-device comparability.**

Response: We now specify, in the procedures, that error metrics (Bias, AE, APE) were computed over the full 20‑minute protocol, including the 5‑minute rest and recovery segments. Reported calories are reported from the devices as total for the duration of the 20-minutes. We recognize that this may differ from the intended use and have added this as a limitation.

2. **Transparency and consistency of data filtering and outlier handling**

**The manuscript refers to “device-specific data quality filters” and the removal of implausible estimates (e.g., values >450% of K5). The conclusions regarding Fitbit accuracy appear particularly sensitive to these decisions. Please provide a clear, device-agnostic description of all filtering rules, indicate whether these criteria were preregistered, and justify any device-specific procedures. I strongly recommend presenting sensitivity analyses with and without outlier removal (at least for Fitbit, ideally for all devices) to demonstrate the robustness of the main findings.**

Response: Our preregistration prespecified the removal of device errors and outlier values, although exact numeric cutpoints were not fully defined in advance. During data review, values that were clearly implausible for the study protocol (≤1 kcal or >450% of the K5 estimate) were operationalized as device-error exclusions and applied uniformly across devices. We have now clarified this in the data analysis section. Because these implausible values occurred disproportionately in Fitbit, the analyzable Fitbit sample was smaller than for the other devices, as we explicitly state in the manuscript. To address transparency, we have added a sensitivity analysis with and without these exclusions to the registration website. These analyses show that retaining the implausible values substantially inflates Fitbit bias, whereas results for the other devices change only modestly. We do report this change in bias briefly in the results section.

3. **Interpretation of skin tone effects**

**Although three methods of assessing skin tone were described, only Fitzpatrick self-report (types III–V) was retained for analysis due to reliability issues with objective measures. Please provide more detail on why colorimetry and SFDS data were excluded. Given the small number of participants with Fitzpatrick type V, the null findings regarding skin tone should be interpreted more cautiously and framed explicitly as limited by statistical power and measurement constraints.**

Response: We appreciate the reviewer’s request for clarification regarding the exclusion of objective skin tone measurements. Although three approaches were initially employed (Fitzpatrick self-assessment, Nix colorimetry, and SFDS), only the Fitzpatrick classification was retained for analysis due to significant reliability and consistency issues with the other methods.

First, the Nix colorimeter, while useful for industrial color matching (e.g., paints), is not designed or validated for human skin measurements. In our study, it produced inconsistent and physiologically implausible readings (e.g., green-biased RGB outputs), indicating poor reliability for biological tissue characterization under our experimental conditions.

Second, SFDS measurements required calibration against a tissue-mimicking optical phantom prior to each acquisition session. Due to multiple researchers collecting data, this calibration step was not consistently performed or documented. Additionally, ambient light contamination, introduced by uncontrolled lighting conditions (e.g., a nearby window or inconsistencies in hallway lighting), further compromised measurement accuracy. There was also inconsistency in acquisition timing relative to participant activity (e.g., pre- vs. post-exercise), which can alter peripheral blood flow and, consequently, the optical properties of the skin. These factors collectively introduced substantial variability and uncertainty into the SFDS-derived metrics.

Given these limitations, we determined that the most consistent and interpretable measure of skin tone in this dataset was the participant-reported Fitzpatrick classification, which is widely used in clinical and biomedical research. Therefore, only self‑reported Fitzpatrick type was retained. A brief summary of this has been added in the method section. Because so few participants had type V skin tone, the null skin‑tone effect is interpreted cautiously, as emphasized in the discussion.

4. **Specification of BF% measurement used in primary analyses**

**Body fat percentage is a central moderator in the models, yet it is unclear which BF% measure (skinfold-derived, BIA-derived, or another composite) was used in the primary analyses. Please clarify this choice and, if feasible, include a brief sensitivity analysis demonstrating whether results are consistent across BF% assessment methods.**

Response: Thank you for this important point. Body fat percentage (BF%) used in the primary analyses was derived from bioelectrical impedance analysis (BIA; Omron HBF-306C), which was selected a priori as the primary indicator due to its standardized protocol, lower operator dependence, and greater consistency across participants relative to skinfold assessment. Skinfold-derived BF% was collected as a secondary/backup measure; however, given its higher susceptibility to inter-rater variability and site-specific measurement error, it was not used in the primary models.

Because both methods aim to estimate the same underlying construct and were strongly aligned in our sample, we prioritized the BIA-derived BF% to maintain consistency and reduce measurement noise in the mixed-effects models. We have clarified this choice in the Methods section .

We agree that sensitivity analyses can be informative; however, given the study’s sample size and the increased measurement variability associated with skinfold estimates, we determined that substituting BF% measures would primarily introduce noise rather than meaningfully alter inference. Our focus was therefore on using the most reliable and standardized adiposity indicator available.

5. **Scope and strength of conclusions**

**While the data clearly demonstrate substantial PAEE error under the tested conditions, some statements regarding the unsuitability of consumer wearables for individual or research use appear overly broad given the single exercise modality and short protocol. I recommend tempering these conclusions and explicitly limiting them to the tested conditions, while distinguishing between absolute caloric accuracy and potential utility for relative or within-person tracking.**

Response: We revised the discussion to state that large absolute errors and adiposity‑related biases were observed during the tested cycling protocol, but emphasise that wearables may still be useful for tracking within‑person changes over time. We also note that our findings may not generalise to other activities or free‑living conditions.

**Minor comments:**

1. **Please confirm that user profiles (age, sex, height, weight) were consistently entered across devices and clarify whether any device incorporated BF% directly.**

Response: We confirm that participant profiles (age, sex, height, weight) were entered identically across all devices to ensure consistency in device-derived estimates. Body‑fat percentage was not entered into any device.

2. **Consider reporting back-transformed effect sizes (e.g., change in APE per 1 SD increase in BF%) to aid interpretation of practical relevance.**

Response: We now report back‑transformed effect sizes to aid interpretation.

3. **Briefly acknowledge the potential influence of wearing multiple devices simultaneously on wrist fit and sensor contact.**

Response: The discussion now notes that wearing multiple devices may affect strap tension and sensor contact. Device placement was counterbalanced across wrists using a Latin‑square design to minimize this effect.

4. **Minor typographical and spacing issues are present and should be corrected during revision.**

Response: The manuscript has been thoroughly proofread to correct typographical and spacing errors.

We thank the editor and reviewers for their constructive feedback. The manuscript has been extensively revised to improve clarity, methodological transparency, and alignment with PLOS ONE guidelines. We hope that the detailed point‑by‑point responses and accompanying revised analyses demonstrate our commitment to rigor and transparency.
