# Supplementary material for: Body fat, skin tone, and the accuracy of smartwatch caloric expenditure estimates
Source: PLoS One. 2026 Jul 29;21(7):e0353261. doi: 10.1371/journal.pone.0353261 (PMC13419227; doi:10.1371/journal.pone.0353261)
Supplement: Peer review history

# Peer review: Decision Letter 0

**DOI:** 10.1371/journal.pone.0353261.r001

**Authors:** Hidetaka Hamasaki

26 Feb 2026

PONE-D-26-00179

Body Fat, Skin Tone, and the Accuracy of Smartwatch Caloric Expenditure Estimates

PLOS One

Dear Dr. Kostrna,

Thank you for submitting your manuscript to PLOS ONE. After careful consideration, we feel that it has merit but does not fully meet PLOS ONE’s publication criteria as it currently stands. Therefore, we invite you to submit a revised version of the manuscript that addresses the points raised during the review process.

Please submit your revised manuscript by Apr 12 2026 11:59PM. If you will need more time than this to complete your revisions, please reply to this message or contact the journal office at plosone@plos.org. When you're ready to submit your revision, log on to https://www.editorialmanager.com/pone/ and select the 'Submissions Needing Revision' folder to locate your manuscript file.

Please include the following items when submitting your revised manuscript:

- A letter that responds to each point raised by the academic editor and reviewer(s). You should upload this letter as a separate file labeled 'Response to Reviewers'.
- A marked-up copy of your manuscript that highlights changes made to the original version. You should upload this as a separate file labeled 'Revised Manuscript with Track Changes'.
- An unmarked version of your revised paper without tracked changes. You should upload this as a separate file labeled 'Manuscript'.

If you would like to make changes to your financial disclosure, please include your updated statement in your cover letter. Guidelines for resubmitting your figure files are available below the reviewer comments at the end of this letter.

If applicable, we recommend that you deposit your laboratory protocols in protocols.io to enhance the reproducibility of your results. Protocols.io assigns your protocol its own identifier (DOI) so that it can be cited independently in the future. For instructions see: https://journals.plos.org/plosone/s/submission-guidelines#loc-laboratory-protocols. Additionally, PLOS ONE offers an option for publishing peer-reviewed Lab Protocol articles, which describe protocols hosted on protocols.io. Read more information on sharing protocols at https://plos.org/protocols?utm_medium=editorial-email&utm_source=authorletters&utm_campaign=protocols.

We look forward to receiving your revised manuscript.

Kind regards,

Hidetaka Hamasaki

Academic Editor

PLOS One

Journal Requirements:

When submitting your revision, we need you to address these additional requirements.

1. Please ensure that your manuscript meets PLOS ONE's style requirements, including those for file naming. The PLOS ONE style templates can be found at

https://journals.plos.org/plosone/s/file?id=wjVg/PLOSOne_formatting_sample_main_body.pdf and

https://journals.plos.org/plosone/s/file?id=ba62/PLOSOne_formatting_sample_title_authors_affiliations.pdf

2. The ethical approval number(s) listed in the manuscript and submission metadata ( # IRB-22-0471-AM03) does not match the approval number on the ethical approval document you provided. Please ensure that all approval numbers are correct.

3. Please note that funding information should not appear in the Acknowledgments section or other areas of your manuscript. We will only publish funding information present in the Funding Statement section of the online submission form. Please remove any funding-related text from the manuscript.

4. Your abstract cannot contain citations. Please only include citations in the body text of the manuscript, and ensure that they remain in ascending numerical order on first mention.

5. If the reviewer comments include a recommendation to cite specific previously published works, please review and evaluate these publications to determine whether they are relevant and should be cited. There is no requirement to cite these works unless the editor has indicated otherwise.

[Note: HTML markup is below. Please do not edit.]

Reviewers' comments:

Reviewer's Responses to Questions

**Comments to the Author**

1. Is the manuscript technically sound, and do the data support the conclusions?

The manuscript must describe a technically sound piece of scientific research with data that supports the conclusions. Experiments must have been conducted rigorously, with appropriate controls, replication, and sample sizes. The conclusions must be drawn appropriately based on the data presented.

Reviewer #1: Yes

Reviewer #2: Partly

**********

2. Has the statistical analysis been performed appropriately and rigorously?

Reviewer #1: Yes

Reviewer #2: Yes

**********

3. Have the authors made all data underlying the findings in their manuscript fully available?

The PLOS Data policy requires authors to make all data underlying the findings described in their manuscript fully available without restriction, with rare exception (please refer to the Data Availability Statement in the manuscript PDF file). The data should be provided as part of the manuscript or its supporting information, or deposited to a public repository. For example, in addition to summary statistics, the data points behind means, medians and variance measures should be available. If there are restrictions on publicly sharing data—e.g. participant privacy or use of data from a third party—those must be specified.

Reviewer #1: Yes

Reviewer #2: Yes

**********

4. Is the manuscript presented in an intelligible fashion and written in standard English?

PLOS ONE does not copyedit accepted manuscripts, so the language in submitted articles must be clear, correct, and unambiguous. Any typographical or grammatical errors should be corrected at revision, so please note any specific errors here.

Reviewer #1: Yes

Reviewer #2: Yes

**********

5. Review Comments to the Author

Please use the space provided to explain your answers to the questions above. You may also include additional comments for the author, including concerns about dual publication, research ethics, or publication ethics. (Please upload your review as an attachment if it exceeds 20,000 characters)

Reviewer #1: Title and Abstract

- Abstract (Lines 50–52): The wording is unclear and would benefit from rephrasing for clarity and flow.

- Physical activity energy expenditure (PAEE) should be defined in full at first mention in the abstract.

- The primary objective is not clearly stated in the abstract and should be explicitly articulated.

- References are generally not included in abstracts; prior work should be described using general phrasing without citation.

Methods

Ethics and Recruitment

- There is a discrepancy between the stated ethics approval date (2024) and the reported recruitment start date (January 2023), which requires clarification.

- The manuscript should explicitly state that participation was voluntary and that alternative assignments were offered to minimise the risk of coercion.

- The recruitment strategy should be clearly planned and described a priori; changes to recruitment methods over time should be justified if applicable.

Participants

- The method used to stratify body mass index (BMI) is not described and should be specified.

- Demographic and baseline characteristics should be reported in the Results section rather than in the Participants subsection of the Methods.

Study Design

- The design is acceptable; however, the authors should briefly clarify the between-subject and within-subject factors of the mixed factorial design and describe the randomisation of device placement conditions.

Measures

- The description of wearable devices is generally clear; however, the authors should clarify wrist placement, ordering of the five devices, and address potential interference from simultaneous wear.

- Ensure that this section is limited to methodological descriptions and does not include results.

Data Analysis

- Line 206 should clearly state whether the COSMED K5 was used as the criterion reference.

- Five devices are described earlier, yet the statistical models refer to device as having four levels; this discrepancy must be clarified.

- The inclusion of all pairwise interaction terms should be briefly justified or stated as prespecified.

- A short explanation should be added to justify the log-transformation of AE and APE.

- Truncated sentences should be corrected and abbreviated terms (for example, “Fitz”) written in full.

- A brief justification for the inclusion of one-sample t-tests alongside mixed-effects models would improve clarity.

Results

- Demographic and baseline participant data should be presented in the Results section.

- While the Results collectively address device- and adiposity-related PAEE error, the authors should ensure that all prespecified objectives, particularly those related to skin tone and the criterion reference, are clearly reflected in the Results structure and subsection headings.

Discussion

- The Discussion is well written and appropriately interprets the findings in light of the study design and analyses. Minor revisions are recommended to more clearly contextualise subgroup limitations and to maintain appropriate caution in device-level comparisons.

Reviewer #2: This manuscript presents a well-designed validation study examining the accuracy of physical activity energy expenditure (PAEE) estimates from four widely used wrist-worn wearables against indirect calorimetry during a controlled cycling protocol. The study is preregistered, employs an appropriate criterion measure (COSMED K5), applies mixed-effects modeling, and makes data and code publicly available via OSF. The focus on body fat percentage (BF%) as a moderator of device error is a clear strength and addresses an important gap in the literature.

However, several methodological and interpretational issues require clarification and additional analyses before the conclusions can be fully supported.

Major concerns:

Clarification of PAEE definition and analyzed time window

The protocol includes rest, exercise, and recovery phases, but it is not fully clear which segments were included in the PAEE error calculations. Please explicitly specify whether error metrics (Bias, AE, APE) were computed over the entire session, the exercise phase only, or separately by intensity. In addition, clarify whether devices reported “active” versus “total” calories and how this was harmonized across brands. These details are critical, as low-intensity periods may disproportionately affect APE and between-device comparability.

Transparency and consistency of data filtering and outlier handling

The manuscript refers to “device-specific data quality filters” and the removal of implausible estimates (e.g., values >450% of K5). The conclusions regarding Fitbit accuracy appear particularly sensitive to these decisions. Please provide a clear, device-agnostic description of all filtering rules, indicate whether these criteria were preregistered, and justify any device-specific procedures. I strongly recommend presenting sensitivity analyses with and without outlier removal (at least for Fitbit, ideally for all devices) to demonstrate the robustness of the main findings.

Interpretation of skin tone effects

Although three methods of assessing skin tone were described, only Fitzpatrick self-report (types III–V) was retained for analysis due to reliability issues with objective measures. Please provide more detail on why colorimetry and SFDS data were excluded. Given the small number of participants with Fitzpatrick type V, the null findings regarding skin tone should be interpreted more cautiously and framed explicitly as limited by statistical power and measurement constraints.

Specification of BF% measurement used in primary analyses

Body fat percentage is a central moderator in the models, yet it is unclear which BF% measure (skinfold-derived, BIA-derived, or another composite) was used in the primary analyses. Please clarify this choice and, if feasible, include a brief sensitivity analysis demonstrating whether results are consistent across BF% assessment methods.

Scope and strength of conclusions

While the data clearly demonstrate substantial PAEE error under the tested conditions, some statements regarding the unsuitability of consumer wearables for individual or research use appear overly broad given the single exercise modality and short protocol. I recommend tempering these conclusions and explicitly limiting them to the tested conditions, while distinguishing between absolute caloric accuracy and potential utility for relative or within-person tracking.

Minor comments:

Please confirm that user profiles (age, sex, height, weight) were consistently entered across devices and clarify whether any device incorporated BF% directly.

Consider reporting back-transformed effect sizes (e.g., change in APE per 1 SD increase in BF%) to aid interpretation of practical relevance.

Briefly acknowledge the potential influence of wearing multiple devices simultaneously on wrist fit and sensor contact.

Minor typographical and spacing issues are present and should be corrected during revision.

Recommendation:

This study meets the technical criteria of PLOS ONE but requires major revision to improve transparency, robustness, and interpretability of the results. Addressing the points above would substantially strengthen the manuscript and its contribution to the literature on wearable-derived energy expenditure estimation.

**********

6. PLOS authors have the option to publish the peer review history of their article (what does this mean?). If published, this will include your full peer review and any attached files.

If you choose “no”, your identity will remain anonymous but your review may still be made public.

**Do you want your identity to be public for this peer review?**  For information about this choice, including consent withdrawal, please see our Privacy Policy.

Reviewer #1: No

Reviewer #2: No

**********

[NOTE: If reviewer comments were submitted as an attachment file, they will be attached to this email and accessible via the submission site. Please log into your account, locate the manuscript record, and check for the action link "View Attachments". If this link does not appear, there are no attachment files.]

To ensure your figures meet our technical requirements, please review our figure guidelines: https://journals.plos.org/plosone/s/figures

You may also use PLOS’s free figure tool, NAAS, to help you prepare publication quality figures: https://journals.plos.org/plosone/s/figures#loc-tools-for-figure-preparation.

NAAS will assess whether your figures meet our technical requirements by comparing each figure against our figure specifications.

# Author comment: Author response to Decision Letter 1

**DOI:** 10.1371/journal.pone.0353261.r002

15 Apr 2026

Ref: PONE-D-26-00179 revision (R1)

Body Fat, Skin Tone, and the Accuracy of Smartwatch Caloric Expenditure Estimates

PLOS One

Editor's comments

Thank you for submitting your manuscript to PLOS ONE. After careful consideration, we feel that it has merit but does not fully meet PLOS ONE’s publication criteria as it currently stands. Therefore, we invite you to submit a revised version of the manuscript that addresses the points raised during the review process.

Response: We thank the Editor and both reviewers for the thoughtful assessment of our manuscript and for the opportunity to revise and improve the work. We appreciate the recognition of the study’s potential contribution and have carefully considered all editor and reviewer comments. The manuscript has been revised extensively to address the suggested improvements and to strengthen clarity, methodological rigor, and interpretation throughout. We detail the resulting changes below and highlight them in the manuscript.

1. Please ensure that your manuscript meets PLOS ONE's style requirements, including those for file naming.

Response: We have reformatted the manuscript in line with the current PLOS ONE templates. Separate files for the main text, and figures have been prepared and named according to the journal's conventions.

2. The ethical approval number(s) listed in the manuscript and submission metadata does not match the approval number on the ethical approval document you provided.

Response: Throughout the manuscript and in the submission metadata we now use the protocol number IRB‑22‑0471, consistent with the approval letter issued by the IRB on 12 October 2022.

3. Funding information should not appear in the Acknowledgments section or other areas of your manuscript.

Response: All funding information has been confined to the Funding Statement within the submission system. The acknowledgments now solely thank the research assistants and study participants.

4. Your abstract cannot contain citations.

Response: The abstract has been rewritten for clarity and now contains no citations. Prior work is referenced in the introduction instead.

5. If the reviewer comments include a recommendation to cite specific previously published works, please review and evaluate these publications to determine whether they are relevant.

Response: There were no directly suggested references.

Reviewer #1

Response: Thank you for your thorough and well-structured review. We have addressed each comment and highlighted the respective changes in the revised manuscript.

Title and Abstract

1. (Lines 50–52): The wording is unclear and would benefit from rephrasing for clarity and flow.

Response: We rewrote the abstract (Lines 50–52) for clarity.

2. Physical activity energy expenditure (PAEE) should be defined in full at first mention in the abstract.

Response: Physical activity energy expenditure (PAEE) is now defined on first use

3. The primary objective is not clearly stated in the abstract and should be explicitly articulated.

Response: The primary objective is explicitly stated: “The primary objective of this study was to quantify the accuracy of smartwatch-derived physical activity energy expenditure (PAEE) estimates relative to indirect calorimetry and to examine whether error varies as a function of device brand, body fat percentage, and skin tone.”

4. References are generally not included in abstracts; prior work should be described using general phrasing without citation.

Response: All citations were removed from the abstract as per journal policy.

Methods

Ethics and Recruitment

5. There is a discrepancy between the stated ethics approval date (2024) and the reported recruitment start date (January 2023), which requires clarification.

Response: The Methods now note that IRB approval was granted on 12 October 2022 and that recruitment began in January 2023 after approval.

6. The manuscript should explicitly state that participation was voluntary and that alternative assignments were offered to minimise the risk of coercion.

Response: We added a statement that participation was voluntary and that course credit–seeking students were offered alternative assignments.

7. The recruitment strategy should be clearly planned and described a priori; changes to recruitment methods over time should be justified if applicable.

Response: The recruitment strategy (campus announcements and flyers) was planned in advance and this is now described in the Procedures section, there are no deviations to report.

Participants

8. The method used to stratify body mass index (BMI) is not described and should be specified.

Response: BMI strata are now described using the World Health Organization cut‑points, and this stratification is only used to characterise the sample.

9. Demographic and baseline characteristics should be reported in the Results section rather than in the Participants subsection of the Methods.

Response: Baseline characteristics (age, sex, BMI, body‑fat percentage, Fitzpatrick skin type) have been moved to the beginning of the Results section.

Study Design

10. The design is acceptable; however, the authors should briefly clarify the between-subject and within-subject factors of the mixed factorial design and describe the randomisation of device placement conditions.

Response: The design is now described as a four‑level within‑subject factor of device (Apple, Garmin, Samsung, Fitbit) crossed with continuous body fat percentage and categorical Fitzpatrick skin type. Device placement was randomized across wrists using a Latin‑square schedule, which is now detailed in the Design and Measures section.

Measures

11. The description of wearable devices is generally clear; however, the authors should clarify wrist placement, ordering of the five devices, and address potential interference from simultaneous wear.

Response: We have clarified the randomization procedure for the devices, included additional details about device placement in the measures section, and previously conducted a sensitivity analysis to search for potential interference (briefly reported in text and fully shared in the OSF registry analysis), which found no evidence of such interference.

12. Ensure that this section is limited to methodological descriptions and does not include results.

Response: We have moved any results to the results section as appropriate.

Data Analysis

13. Line 206 should clearly state whether the COSMED K5 was used as the criterion reference.

Response: We have modified the language to explicitly state that the COSMED K5 served as the criterion reference.

14. Five devices are described earlier, yet the statistical models refer to device as having four levels; this discrepancy must be clarified.

Response: The Empatica E4 did not report caloric expenditure and was therefore excluded from analyses as indicated and now clarified in the measures section, leaving four device levels. Originally, the Empatica was included for its ability to provide open-source access to the PPG waveform; software support for the device ended before analysis.

15. The inclusion of all pairwise interaction terms should be briefly justified or stated as prespecified.

Response: All pairwise interactions among device, body‑fat percentage (z‑scored), and Fitzpatrick type were prespecified and this is now explained.

16. A short explanation should be added to justify the log-transformation of AE and APE.

Response: Log‑transformation of absolute error (AE) and absolute percentage error (APE) is justified to normalize right‑skewed distributions, this has been explained in text.

17. Truncated sentences should be corrected and abbreviated terms (for example, “Fitz”) written in full.

Response: Abbreviations such as ‘Fitz’ have been replaced with full terms and the truncated sentences have been corrected.

18. A brief justification for the inclusion of one-sample t-tests alongside mixed-effects models would improve clarity.

Response: One‑sample t‑tests are included to assess whether mean device bias differs from zero prior to mixed‑effects modelling, as clarified in the Data Analysis section.

Results

19. Demographic and baseline participant data should be presented in the Results section.

Response: Demographic and baseline characteristics now open the Results section.

20. While the Results collectively address device- and adiposity-related PAEE error, the authors should ensure that all prespecified objectives, particularly those related to skin tone and the criterion reference, are clearly reflected in the Results structure and subsection headings.

Response: The Results are have been reorganized into subsections which contain clear outcomes related to prespecified objectives (device error metric, body fat effects, skin tone effects and criterion reference performance) to ensure that each is clearly addressed.

Discussion

21. The Discussion is well written and appropriately interprets the findings in light of the study design and analyses. Minor revisions are recommended to more clearly contextualise subgroup limitations and to maintain appropriate caution in device-level comparisons.

Response: Thank you for this compliment. We added a sentnce noting that the sample comprised primarily Hispanic adults with Fitzpatrick types III–V, and that the null skin‑tone finding should be interpreted cautiously due to limited statistical power.

Reviewer #2

This manuscript presents a well-designed validation study examining the accuracy of physical activity energy expenditure (PAEE) estimates from four widely used wrist-worn wearables against indirect calorimetry during a controlled cycling protocol. The study is preregistered, employs an appropriate criterion measure (COSMED K5), applies mixed-effects modeling, and makes data and code publicly available via OSF. The focus on body fat percentage (BF%) as a moderator of device error is a clear strength and addresses an important gap in the literature.

However, several methodological and interpretational issues require clarification and additional analyses before the conclusions can be fully supported.

Response: Thank you for your review and the recognition of the strengths of this research as well as your thorough critique. We have addressed each comment and highlighted the respective changes in the revised manuscript.

Major concerns:

1. Clarification of PAEE definition and analyzed time window

The protocol includes rest, exercise, and recovery phases, but it is not fully clear which segments were included in the PAEE error calculations. Please explicitly specify whether error metrics (Bias, AE, APE) were computed over the entire session, the exercise phase only, or separately by intensity. In addition, clarify whether devices reported “active” versus “total” calories and how this was harmonized across brands. These details are critical, as low-intensity periods may disproportionately affect APE and between-device comparability.

Response: We now specify, in the procedures, that error metrics (Bias, AE, APE) were computed over the full 20‑minute protocol, including the 5‑minute rest and recovery segments. Reported calories are reported from the devices as total for the duration of the 20-minutes. We recognize that this may differ from the intended use and have added this as a limitation.

2. Transparency and consistency of data filtering and outlier handling

The manuscript refers to “device-specific data quality filters” and the removal of implausible estimates (e.g., values >450% of K5). The conclusions regarding Fitbit accuracy appear particularly sensitive to these decisions. Please provide a clear, device-agnostic description of all filtering rules, indicate whether these criteria were preregistered, and justify any device-specific procedures. I strongly recommend presenting sensitivity analyses with and without outlier removal (at least for Fitbit, ideally for all devices) to demonstrate the robustness of the main findings.

Response: Our preregistration prespecified the removal of device errors and outlier values, although exact numeric cutpoints were not fully defined in advance. During data review, values that were clearly implausible for the study protocol (≤1 kcal or >450% of the K5 estimate) were operationalized as device-error exclusions and applied uniformly across devices. We have now clarified this in the data analysis section. Because these implausible values occurred disproportionately in Fitbit, the analyzable Fitbit sample was smaller than for the other devices, as we explicitly state in the manuscript. To address transparency, we have added a sensitivity analysis with and without these exclusions to the registration website. These analyses show that retaining the implausible values substantially inflates Fitbit bias, whereas results for the other devices change only modestly. We do report this change in bias briefly in the results section.

3. Interpretation of skin tone effects

Although three methods of assessing skin tone were described, only Fitzpatrick self-report (types III–V) was retained for analysis due to reliability issues with objective measures. Please provide more detail on why colorimetry and SFDS data were excluded. Given the small number of participants with Fitzpatrick type V, the null findings regarding skin tone should be interpreted more cautiously and framed explicitly as limited by statistical power and measurement constraints.

Response: We appreciate the reviewer’s request for clarification regarding the exclusion of objective skin tone measurements. Although three approaches were initially employed (Fitzpatrick self-assessment, Nix colorimetry, and SFDS), only the Fitzpatrick classification was retained for analysis due to significant reliability and consistency issues with the other methods.

First, the Nix colorimeter, while useful for industrial color matching (e.g., paints), is not designed or validated for human skin measurements. In our study, it produced inconsistent and physiologically implausible readings (e.g., green-biased RGB outputs), indicating poor reliability for biological tissue characterization under our experimental conditions.

Second, SFDS measurements required calibration against a tissue-mimicking optical phantom prior to each acquisition session. Due to multiple researchers collecting data, this calibration step was not consistently performed or documented. Additionally, ambient light contamination, introduced by uncontrolled lighting conditions (e.g., a nearby window or inconsistencies in hallway lighting), further compromised measurement accuracy. There was also inconsistency in acquisition timing relative to participant activity (e.g., pre- vs. post-exercise), which can alter peripheral blood flow and, consequently, the optical properties of the skin. These factors collectively introduced substantial variability and uncertainty into the SFDS-derived metrics.

Given these limitations, we determined that the most consistent and interpretable measure of skin tone in this dataset was the participant-reported Fitzpatrick classification, which is widely used in clinical and biomedical research. Therefore, only self‑reported Fitzpatrick type was retained. A brief summary of this has been added in the method section. Because so few participants had type V skin tone, the null skin‑tone effect is interpreted cautiously, as emphasized in the discussion.

4. Specification of BF% measurement used in primary analyses

Body fat percentage is a central moderator in the models, yet it is unclear which BF% measure (skinfold-derived, BIA-derived, or another composite) was used in the primary analyses. Please clarify this choice and, if feasible, include a brief sensitivity analysis demonstrating whether results are consistent across BF% assessment methods.

Response: Thank you for this important point. Body fat percentage (BF%) used in the primary analyses was derived from bioelectrical impedance analysis (BIA; Omron HBF-306C), which was selected a priori as the primary indicator due to its standardized protocol, lower operator dependence, and greater consistency across participants relative to skinfold assessment. Skinfold-derived BF% was collected as a secondary/backup measure; however, given its higher susceptibil

Attachment Submitted filename: reviwers comments PLOS ONE R1 PONE-D-26-00179.docx

# Peer review: Decision Letter 1

**DOI:** 10.1371/journal.pone.0353261.r003

**Authors:** Hidetaka Hamasaki, Hidetaka Hamasaki

11 Jun 2026

PONE-D-26-00179R1

Body fat, skin tone, and the accuracy of smartwatch caloric expenditure estimates

PLOS One

Dear Dr. Kostrna,

Thank you for submitting your manuscript to PLOS ONE. After careful consideration, we feel that it has merit but does not fully meet PLOS ONE’s publication criteria as it currently stands. Therefore, we invite you to submit a revised version of the manuscript that addresses the points raised during the review process.

==============================

**ACADEMIC EDITOR:**

Thank you for submitting the revised manuscript. It seems that some sections may still require additional revision. Please carefully address the reviewers' comments and resubmit the manuscript at your convenience.

==============================

Please submit your revised manuscript by Jul 26 2026 11:59PM. If you will need more time than this to complete your revisions, please reply to this message or contact the journal office at plosone@plos.org. When you're ready to submit your revision, log on to https://www.editorialmanager.com/pone/ and select the 'Submissions Needing Revision' folder to locate your manuscript file.

Please include the following items when submitting your revised manuscript:

- A letter that responds to each point raised by the academic editor and reviewer(s). You should upload this letter as a separate file labeled 'Response to Reviewers'.
- A marked-up copy of your manuscript that highlights changes made to the original version. You should upload this as a separate file labeled 'Revised Manuscript with Track Changes'.
- An unmarked version of your revised paper without tracked changes. You should upload this as a separate file labeled 'Manuscript'.

If you would like to make changes to your financial disclosure, please include your updated statement in your cover letter. Guidelines for resubmitting your figure files are available below the reviewer comments at the end of this letter.

If applicable, we recommend that you deposit your laboratory protocols in protocols.io to enhance the reproducibility of your results. Protocols.io assigns your protocol its own identifier (DOI) so that it can be cited independently in the future. For instructions see: https://journals.plos.org/plosone/s/submission-guidelines#loc-laboratory-protocols. Additionally, PLOS ONE offers an option for publishing peer-reviewed Lab Protocol articles, which describe protocols hosted on protocols.io. Read more information on sharing protocols at https://plos.org/protocols?utm_medium=editorial-email&utm_source=authorletters&utm_campaign=protocols.

As the corresponding author, your ORCID iD is verified in the submission system and will appear in the published article. PLOS supports the use of ORCID, and we encourage all coauthors to register for an ORCID iD and use it as well. Please encourage your coauthors to verify their ORCID iD within the submission system before final acceptance, as unverified ORCID iDs will not appear in the published article. *Only*  the individual author can complete the verification step; PLOS staff *cannot*  verify ORCID iDs on behalf of authors.

We look forward to receiving your revised manuscript.

Kind regards,

Hidetaka Hamasaki

Academic Editor

PLOS One

**Journal Requirements:**

1. If the reviewer comments include a recommendation to cite specific previously published works, please review and evaluate these publications to determine whether they are relevant and should be cited. There is no requirement to cite these works unless the editor has indicated otherwise.

2. Please review your reference list to ensure that it is complete and correct. If you have cited papers that have been retracted, please include the rationale for doing so in the manuscript text, or remove these references and replace them with relevant current references. Any changes to the reference list should be mentioned in the rebuttal letter that accompanies your revised manuscript. If you need to cite a retracted article, indicate the article’s retracted status in the References list and also include a citation and full reference for the retraction notice.

[Note: HTML markup is below. Please do not edit.]

Reviewers' comments:

Reviewer's Responses to Questions

**Comments to the Author**

1. If the authors have adequately addressed your comments raised in a previous round of review and you feel that this manuscript is now acceptable for publication, you may indicate that here to bypass the “Comments to the Author” section, enter your conflict of interest statement in the “Confidential to Editor” section, and submit your "Accept" recommendation.

Reviewer #3: All comments have been addressed

**********

2. Is the manuscript technically sound, and do the data support the conclusions?

The manuscript must describe a technically sound piece of scientific research with data that supports the conclusions. Experiments must have been conducted rigorously, with appropriate controls, replication, and sample sizes. The conclusions must be drawn appropriately based on the data presented.

Reviewer #3: Yes

**********

3. Has the statistical analysis been performed appropriately and rigorously?

Reviewer #3: Yes

**********

4. Have the authors made all data underlying the findings in their manuscript fully available?

The PLOS Data policy requires authors to make all data underlying the findings described in their manuscript fully available without restriction, with rare exception (please refer to the Data Availability Statement in the manuscript PDF file). The data should be provided as part of the manuscript or its supporting information, or deposited to a public repository. For example, in addition to summary statistics, the data points behind means, medians and variance measures should be available. If there are restrictions on publicly sharing data—e.g. participant privacy or use of data from a third party—those must be specified.

Reviewer #3: Yes

**********

5. Is the manuscript presented in an intelligible fashion and written in standard English?

PLOS ONE does not copyedit accepted manuscripts, so the language in submitted articles must be clear, correct, and unambiguous. Any typographical or grammatical errors should be corrected at revision, so please note any specific errors here.

Reviewer #3: Yes

**********

6. Review Comments to the Author

Please use the space provided to explain your answers to the questions above. You may also include additional comments for the author, including concerns about dual publication, research ethics, or publication ethics. (Please upload your review as an attachment if it exceeds 20,000 characters)

**Reviewer #3:** I believe the authors have already successfully addressed the comments raised by previous reviewers, and the manuscript is suitable for publication. I would only suggest briefly indicating the BMI levels/cut-offs according to WHO classification standards for clarity.

**********

7. PLOS authors have the option to publish the peer review history of their article (what does this mean?). If published, this will include your full peer review and any attached files.

If you choose “no”, your identity will remain anonymous but your review may still be made public.

**Do you want your identity to be public for this peer review?**  For information about this choice, including consent withdrawal, please see our Privacy Policy.

Reviewer #3: **Yes:** Dr Encarna Micó Amigo

**********

[NOTE: If reviewer comments were submitted as an attachment file, they will be attached to this email and accessible via the submission site. Please log into your account, locate the manuscript record, and check for the action link "View Attachments". If this link does not appear, there are no attachment files.]

To ensure your figures meet our technical requirements, please review our figure guidelines: https://journals.plos.org/plosone/s/figures

You may also use PLOS’s free figure tool, NAAS, to help you prepare publication quality figures: https://journals.plos.org/plosone/s/figures#loc-tools-for-figure-preparation.

NAAS will assess whether your figures meet our technical requirements by comparing each figure against our figure specifications.

# Author comment: Author response to Decision Letter 2

**DOI:** 10.1371/journal.pone.0353261.r004

12 Jun 2026

Reviewers' comments:

Reviewer's Responses to Questions

Comments to the Author

1. If the authors have adequately addressed your comments raised in a previous round of review and you feel that this manuscript is now acceptable for publication, you may indicate that here to bypass the “Comments to the Author” section, enter your conflict of interest statement in the “Confidential to Editor” section, and submit your "Accept" recommendation.

Reviewer #3: All comments have been addressed

Response: Thank you.

________________________________________

2. Is the manuscript technically sound, and do the data support the conclusions?

The manuscript must describe a technically sound piece of scientific research with data that supports the conclusions. Experiments must have been conducted rigorously, with appropriate controls, replication, and sample sizes. The conclusions must be drawn appropriately based on the data presented.

Reviewer #3: Yes

Response: Thank you.

________________________________________

3. Has the statistical analysis been performed appropriately and rigorously?

Reviewer #3: Yes

Response: Thank you.

________________________________________

4. Have the authors made all data underlying the findings in their manuscript fully available?

The PLOS Data policy requires authors to make all data underlying the findings described in their manuscript fully available without restriction, with rare exception (please refer to the Data Availability Statement in the manuscript PDF file). The data should be provided as part of the manuscript or its supporting information, or deposited to a public repository. For example, in addition to summary statistics, the data points behind means, medians and variance measures should be available. If there are restrictions on publicly sharing data—e.g. participant privacy or use of data from a third party—those must be specified.

Reviewer #3: Yes

Response: Thank you.

________________________________________

5. Is the manuscript presented in an intelligible fashion and written in standard English?

PLOS ONE does not copyedit accepted manuscripts, so the language in submitted articles must be clear, correct, and unambiguous. Any typographical or grammatical errors should be corrected at revision, so please note any specific errors here.

Reviewer #3: Yes

Response: Thank you.

________________________________________

6. Review Comments to the Author

Please use the space provided to explain your answers to the questions above. You may also include additional comments for the author, including concerns about dual publication, research ethics, or publication ethics. (Please upload your review as an attachment if it exceeds 20,000 characters)

Reviewer #3: I believe the authors have already successfully addressed the comments raised by previous reviewers, and the manuscript is suitable for publication. I would only suggest briefly indicating the BMI levels/cut-offs according to WHO classification standards for clarity.

Response: We have revised this sentence to indicate the WHO categorization of BMI for obesity as 30.0 kg/m2.

Attachment Submitted filename: reviwers comments PLOS ONE R2 PONE-D-26-00179.docx

# Editor report: Decision Letter 2

**DOI:** 10.1371/journal.pone.0353261.r005

**Authors:** Hidetaka Hamasaki, Hidetaka Hamasaki, Hidetaka Hamasaki

22 Jun 2026

Body fat, skin tone, and the accuracy of smartwatch caloric expenditure estimates

PONE-D-26-00179R2

Dear Dr. Kostrna,

We’re pleased to inform you that your manuscript has been judged scientifically suitable for publication and will be formally accepted for publication once it meets all outstanding technical requirements.

Within one week, you’ll receive an e-mail detailing the required amendments. When these have been addressed, you’ll receive a formal acceptance letter and your manuscript will be scheduled for publication.

An invoice will be generated when your article is formally accepted. Please note, if your institution has a publishing partnership with PLOS and your article meets the relevant criteria, all or part of your publication costs will be covered. Please make sure your user information is up-to-date by logging into Editorial Manager at Editorial Manager® and clicking the ‘Update My Information' link at the top of the page. For questions related to billing, please contact billing support.

If your institution or institutions have a press office, please notify them about your upcoming paper to help maximize its impact. If they’ll be preparing press materials, please inform our press team as soon as possible -- no later than 48 hours after receiving the formal acceptance. Your manuscript will remain under strict press embargo until 2 pm Eastern Time on the date of publication. For more information, please contact onepress@plos.org.

Kind regards,

Hidetaka Hamasaki

Academic Editor

PLOS One

Additional Editor Comments (optional):

Reviewers' comments:

# Editor report: Acceptance letter

**DOI:** 10.1371/journal.pone.0353261.r006

**Authors:** Hidetaka Hamasaki, Hidetaka Hamasaki, Hidetaka Hamasaki

PONE-D-26-00179R2

PLOS One

Dear Dr. Kostrna,

I'm pleased to inform you that your manuscript has been deemed suitable for publication in PLOS One. Congratulations! Your manuscript is now being handed over to our production team.

At this stage, our production department will prepare your paper for publication. This includes ensuring the following:

* All references, tables, and figures are properly cited

* All relevant supporting information is included in the manuscript submission,

* There are no issues that prevent the paper from being properly typeset

You will receive further instructions from the production team, including instructions on how to review your proof when it is ready. Please keep in mind that we are working through a large volume of accepted articles, so please give us a few days to review your paper and let you know the next and final steps.

Lastly, if your institution or institutions have a press office, please let them know about your upcoming paper now to help maximize its impact. If they'll be preparing press materials, please inform our press team within the next 48 hours. Your manuscript will remain under strict press embargo until 2 pm Eastern Time on the date of publication. For more information, please contact onepress@plos.org.

You will receive an invoice from PLOS for your publication fee after your manuscript has reached the completed accept phase. If you receive an email requesting payment before acceptance or for any other service, this may be a phishing scheme. Learn how to identify phishing emails and protect your accounts at https://explore.plos.org/phishing.

If we can help with anything else, please email us at customercare@plos.org.

Thank you for submitting your work to PLOS ONE and supporting open access.

Kind regards,

PLOS ONE Editorial Office Staff

on behalf of

Dr. Hidetaka Hamasaki

Academic Editor

PLOS One
